# Crystallization Behavior of Al_70_Fe_12.5_V_12.5_Nb_5_ Amorphous Alloy Formed by Mechanical Alloying

**DOI:** 10.3390/ma12030383

**Published:** 2019-01-26

**Authors:** Xuan Liu, Xingfu Wang, Yongli Si, Xiaokang Zhong, Fusheng Han

**Affiliations:** 1 Key Laboratory of Materials Physics, Institute of Solid State Physics, Chinese Academy of Science, Hefei 230031, China; liuxuan@ahjzu.edu.cn (X.L.); wangxingfu@issp.ac.cn (X.W.); siyongli@mail.ustc.edu.cn (Y.S.); xkzhong@mail.ustc.edu.cn (X.Z.); 2 University of Science and Technology of China, Hefei 230026, China; 3 College of Civil Engineering, Anhui Jianzhu University, Hefei 230061, China

**Keywords:** aluminum-based amorphous alloys, mechanical alloying, supercooled liquid region, Nb addition

## Abstract

In this study, the formation and crystallization of the Al_70_Fe_12.5_V_12.5_Nb_5_ amorphous alloys has been investigated. The addition of Nb enhances the supercooled liquid region and glass forming ability of the Al-Fe-V amorphous alloys. The Al_70_Fe_12.5_V_12.5_Nb_5_ amorphous alloy exhibits two distinct crystallization steps and a large supercooled liquid region at more than 100 K. Kissinger and Ozawa analyses showed that the two activation energies for crystallization (E_x_) were estimated to be 366.3 ± 23.9 and 380.5 ± 23.9 kJ/mol. Large supercooled liquid regions are expected to gain an application field of Al-based amorphous alloys.

## 1. Introduction

Currently, the application of amorphous powder in bulk metallic glasses, composite, coating, and 3D printing has widened the application scope of amorphous alloys [1,2,3,4]. In particular, Aluminum-based amorphous alloys have attracted increasing attention due to their exceptional physical properties, low glass forming ability (GFA), and poor thermal stability [5,6,7]. However, Al-based amorphous alloys with low GFA are challenging to be synthesized via the traditional rapid quenching (RQ). The size factor restricts the practical application of Aluminum-based amorphous alloys. Indeed, up to now, the maximum diameter of an Al-based amorphous alloys sample is 2.5 mm by RQ [8]. Mechanical alloying (MA) is an effective process to produce amorphous alloy powders without a high cooling rate [9,10]. Therefore, it is noteworthy to prepare Al-based amorphous powders via MA to widen their application [11,12,13,14,15]. To use amorphous powder, a large supercooled liquid region (ΔT_x_) is necessary, and enhancement of the GFA and thermal stability is required.

The addition of Nb can improve the GFA, thermal stability, and the corrosion resistance of Zr-based Zr-Ni-Al-Nb bulk metallic glasses with high compressive yield strength [16]. A similar phenomenon has been observed in some Al-based amorphous alloys [17,18]. It has been reported that, in the case of the Nb-Al system, the sequence of phase formation varies widely depending upon Al content: for example, Al_80_Fe_10_Nb_10_ created via an MA process lasted 100 h is a powder mixture [19,20]. However, according to the efficient cluster packing (ECP) model, Nb has the same atomic radius as Al, so they can be mixed with an arbitrary ratio to occupy the Ω position of the solvent atoms without changing the cluster packing structure in the amorphous alloys [21]. Thus, it is interesting to investigate the GFA and thermal stability of in Al-based amorphous alloys in which Nb has replaced Al. Al_75_V_12.5_Fe_12.5_ amorphous alloys with high GFA and thermal stability were prepared via MA and based on the ECP model [22]. In general, a negative heating of the mixing enthalpy (ΔH_mix_) among elements in the amorphous alloy is beneficial for enhancing the GFA [23]. Nb contribution to the enthalpy leads to a negative ΔH_mix_ with Al (−18 kJ/mol), Fe (−16 kJ/mol), and V (−1 kJ/mol) [24]. Therefore, the GFA and thermal stability expect to be enhanced with the Nb addition in the Al-Fe-V alloys. In this work, we synthesized alloys with 5% of Al replaced by Nb. The GFA and thermal stability were investigated according to the values obtained from differential scanning calorimetry (DSC). The aim of this study is to obtain Al-based amorphous powder with high ΔT_x_ and thermal stability for industrial applications.

## 2. Experimental Details

The Al_70_Fe_12.5_V_12.5_Nb_5_ (at %) amorphous alloy powders were prepared via MA of a mixture of elemental Al (99.9%), Fe (99.8%), V (99.9%), and Nb (99.7%) powders. Stearic acid powder (2 wt. %) was used as the process control agent. The MA was conducted in a high-energy planetary ball mill (QM-3SP4, Instrument Factory of Nanjing University, Nanjing, China) using a stainless steel vial and a steel ball bearing (GGr15 with diameters of 20 mm, 10 mm, and 5 mm are mixed) with the rotation speed of 350 rpm and ball-to-powder weight ratio of 20:1 in a highly pure Ar atmosphere. The vial is introduced into the vacuum glove box every 10 h to obtain the powder samples for analysis. The powder samples were characterized via X-ray diffraction (XRD, X’ Pert Pro MPD, Philips) using Cu Ka radiation in the 2θ range of 20° to 90°. The X’Pert HighScore Plus software (Version: 3.0) is used to analyze the XRD curves of the mixed powder after ball milling. The microstructures of the amorphous powders were examined using transmission electron microscopy (TEM, JEOL 2010, JEOL) and selected area electron diffraction (SAED) operating at 200 kV. The thermal properties, such as T_g_ (the glass transition temperature), T_x_ (the onset crystallization temperature), T_p1_ (the first crystallization peak temperature), T_p2_ (the secondary crystallization peak temperature), T_m_ (the melting temperature), T_l_ (the liquidus temperature), were measured via the DSC (NETZSCH DSC 404F3) at heating rates of 10, 20, 30, 40 K/min from 303 K to 1473 K in an Ar atmosphere.

## 3. Results and Discussion

**Figure 1:**
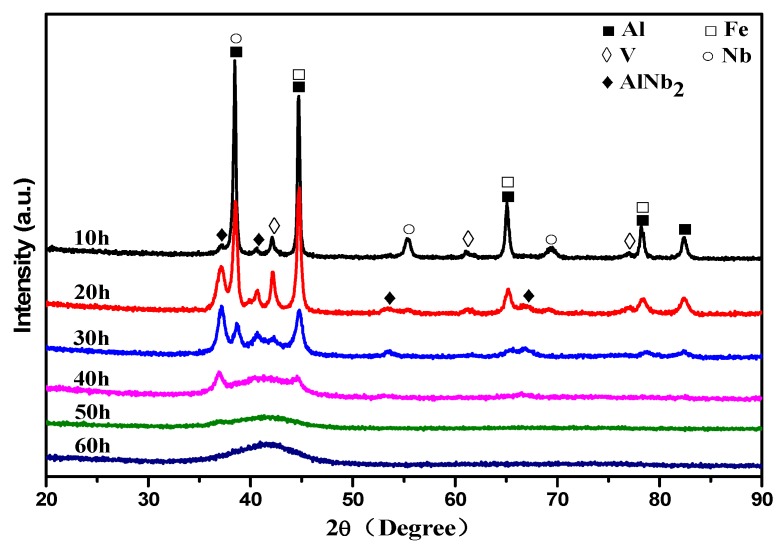
X-ray diffraction patterns (XRD) patterns of Al_70_Fe_12.5_V_12.5_Nb_5_ powders milled for 10, 20, 30, 40, 50 and 60 h.

XRD results of Al_70_Fe_12.5_V_12.5_Nb_5_ alloy powders milled for different periods (10 h, 20 h, 30 h, 40 h, 50 h and 60 h) are shown in Figure 1. It can be seen from Figure 1 that the crystalline phase is formed by ball milling 10 h, X’Pert HighScore Plus software results suggest that the crystalline phase is AlNb_2_. With the increase of milling time, no new diffraction peaks have been found in the XRD curve, indicating that no new crystalline phase is formed except for AlNb_2_. From the DSC curve of 10 h to 30 h, the intensity of the diffraction peak corresponding to the initial addition of powder elements is reduced with the strongest diffraction peak position moving towards higher 2θ angles of 0.2°. According to the Scherrer formula, this means that the lattice of Al has infiltrated other atoms with a smaller atomic radius and formed a solid solution. The atomic radius of Fe (0.124 nm) and V (0.132 nm) is shorter than the radius of Al (0.143 nm); therefore, the product is the Al-Fe-V solid solution formed by Fe and V dissolved in Al. When milling to 40 h, the diffraction peak still exists and has broadened, indicating that the amorphous phase is formed and there are crystalline phases and solid solutions at this time. After milling 50 h, the diffraction peaks of all crystals disappear, while different diffuse peaks can be identified at a 2θ angle of 37°. Until the 60 h milling treatment, only the typical broad halo peak showing the amorphous structure exists, indicating that the powders are amorphous.

**Figure 2:**
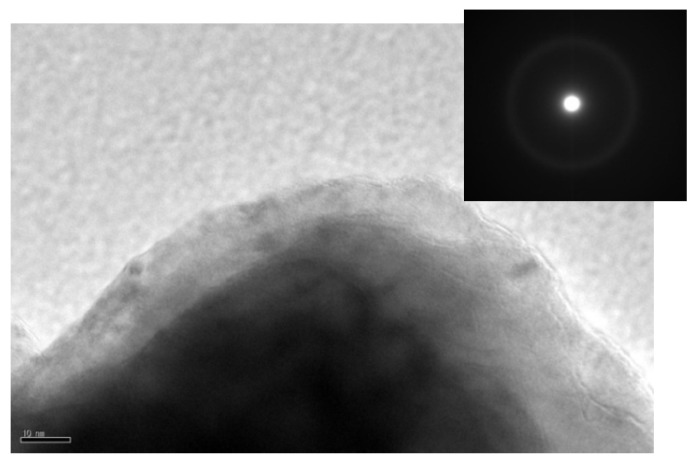
Transmission electron microscope (TEM) image and selected area electron diffraction (SAED) pattern of Al_70_Fe_12.5_V_12.5_Nb_5_ alloys powder milled for 60 h.

To verify the amorphous structure, the TEM and selected area electron diffraction (SEAD) images of Al_70_Fe_12.5_V_12.5_Nb_5_ alloys powder milled for 60 h are shown in Figure 2. There is no visible crystals in the TEM image, and the broad diffuse halo and the absence of rings or diffraction spots in the SEAD pattern further confirms the amorphous structure.

The GFA and thermal stability of the amorphous alloy are related to atomic radii mismatch and thermodynamics of the alloy systems: it can be displayed by the milling time of MA amorphous powders. Compared with the Al_75_V_12.5_Fe_12.5−*x*_Cu*_x_* amorphous alloys reported in the literature [22], the Al_70_Fe_12.5_V_12.5_Nb_5_ amorphous alloy milling time is reduced.

The parameter *Delta* is adopted to describe the effect of atomic radii mismatch of multi-component alloys as follows [25]:

(1) $$Delta=\sqrt{\sum_{i=1}^{n}c_{i}{\left(1-r_{i}/\bar{r}\right)}^{2}}.$$

Here, the *n* is the number of components in the amorphous alloy system, $c_{i}$ is the atomic percentage of the *i* component, and $r_{i}$ is the atomic radius, $\bar{r}\;(\bar{r}=\sum_{i=1}^{n}c_{i}r_{i})$ is the average atomic radius.

By calculating the *Delta*, the Al_70_Fe_12.5_V_12.5_Nb_5_ and Al_75_V_12.5_Fe_12.5−*x*_Cu*_x_* amorphous alloys have the same value. We can then infer that the influence of atomic radii mismatch of the two systems can be neglected.

Another parameter, the equation of the Gibbs free energy ($∆G_{mix}$), is used to characterize the thermodynamics of alloy systems.

(2) $$∆G_{mix}=∆H_{mix}-T∆S_{mix}.$$

According to Equation (2), a low mixing enthalpy ($∆H_{mix}$) or a high entropy of mixing ($∆S_{mix}$) can decrease the $∆G_{mix}$, thus lowering the crystallization driving force, which consequently renders the formation of the amorphous phase easier. $∆H_{mix}$ and $∆S_{mix}$ can be calculated by Equations (3) and (4), respectively.

(3) $$∆H_{mix}=\sum_{i=1,i\neq j}^{n}\Omega_{ij}c_{i}c_{j},$$

(4) $$∆S_{mix}=-R\sum_{i=1,i\neq j}^{n}c_{i}Lnc_{i},$$

where $\Omega_{ij}=4∆H_{ij}$, the $∆H_{ij}$ is the mixing enthalpy of the *i* and *j* elements, and R is the gas constant. It can be concluded that the $∆H_{mix}$ increases in the order of the Al_70_Fe_12.5_V_12.5_Nb_5_ (−11.970 kJ/mol), Al_75_V_12.5_Fe_12.5_ (−10.125 kJ/mol), Al_75_V_12.5_Fe_6.25_Cu_6.25_ (−8.250 kJ/mol), Al_75_V_12.5_Cu_12.5_ (−6.375 kJ/mol) amorphous alloys. The $∆S_{mix}$ decreases in the order of the Al_70_Fe_12.5_V_12.5_Nb_5_ (0.919R), Al_75_V_12.5_Fe_12.5_ (0.736R), Al_75_V_12.5_Cu_12.5_ (0.736R), Al_75_V_12.5_Fe_6.25_Cu_6.25_ (0.649R) amorphous alloys. In these amorphous alloys, Al_70_Fe_12.5_V_12.5_Nb_5_ has the lowest $∆H_{mix}$ and the highest $∆S_{mix}$, so the $∆G_{mix}$ is the lowest according to Equation (2). This may be the reason why the Al_70_Fe_12.5_V_12.5_Nb_5_ amorphous alloy milling time is shortest among these amorphous alloys. Thus, we conclude that the GFA and thermal stability of Al_70_Fe_12.5_V_12.5_Nb_5_ amorphous alloy are higher than Al_75_V_12.5_Fe_12.5−*x*_Cu*_x_* amorphous alloys.

**Figure 3:**
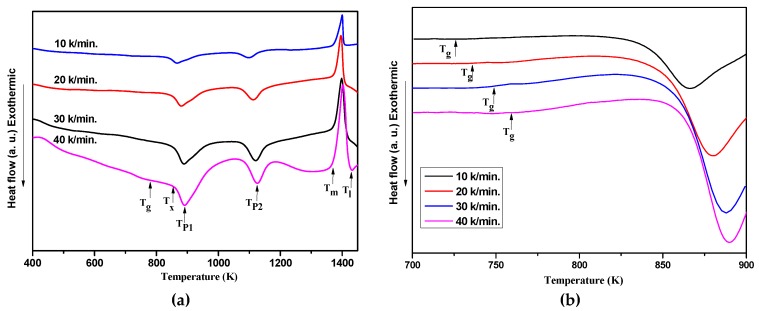
(**a**) Differential scanning calorimetry (DSC) curves of the Al_70_Fe_12.5_V_12.5_Nb_5_ amorphous alloy at the heating rates of 10, 20, 30, and 40 K/min. (**b**) Enlarged area of the DSC curves shown in (**a**) of the T_g_.

Figure 3a shows the DSC curves of the Al_70_Fe_12.5_V_12.5_Nb_5_ amorphous alloy at the heating rates of 10, 20, 30, and 40 K/min. Two exothermic crystallization events can be observed and are associated with the transformations from an amorphous state to the equilibrium phases. The temperature of T_g_ that indicates glass transition at the different heating rate (see Figure 3b). The thermal properties of T_g_, T_x_, T_p1_, T_p2_, T_m_, T_l_ are marked with arrows in Figure 3a and listed in Table 1, which also contains the empirical criteria of GFA [23,26], ΔT_x_ (ΔT_x_ = T_x_ − T_g_), the reduced glass transition temperature T_rg_(T_rg_ = T_g_/T_l_), the γ val (γ = T_x_/(T_g_ + T_l_)). It is clear that, with the increase of heating rate, T_g_, T_x_, T_p1_, T_p2_, turn to a higher temperature, while the ΔT_x_ fluctuates between 106.5 and 117.5 K. This shows that the thermal properties of Al_70_Fe_12.5_V_12.5_Nb_5_ at different heating rates are influenced by kinetic factors. The values of T_x_ and ΔT_x_ of Al_70_Fe_12.5_V_12.5_Nb_5_ amorphous alloy are 854.1 K and 118.6 K at the heating rates of 20 K/min, respectively, whereas the T_m_ is almost unchanged. It should be noted that the change of ΔT_x_ for Al_70_Fe_12.5_V_12.5_Nb_5_ is more than 100 K, which is higher than Al_75_V_12.5_Fe_12.5−*x*_Cu*_x_* amorphous alloys and other Al-based amorphous alloys [27]. The higher ΔT_x_,T_rg_, γ indicates that the Al_70_Fe_12.5_V_12.5_Nb_5_ amorphous alloy has a higher glass forming ability. Therefore, it is a promising candidate for thermoplastic forming BMGs and composite materials via the consolidation of amorphous powders.

The activation energies of E_x_, E_p1_, E_p2_ corresponding to T_x_, T_p1_, T_p2_, respectively, can be estimated via the equations of Kissinger (Equation (5)) and Ozawa (Equation (6)) [28,29]:

(5) $$\mathrm{Ln}\;\left(\beta /T^{2}\right)=-\left(E/\mathrm{RT}\right)+\mathrm{Const},$$

(6) Ln (β)=−(E/RT)+Const.

Here, the β is the heating rates, T represents the specific temperature (T_x_, T_p1_, T_p2_), E is the corresponding activation energy (E_x_, E_p1_, E_p2_), and R is the gas constant.

**Figure 4:**
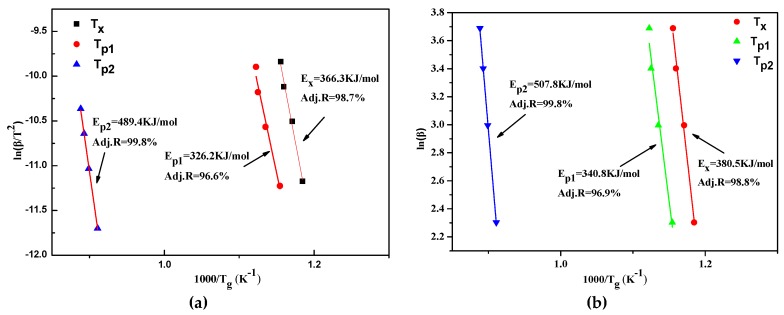
The Kissinger and Ozawa plots of T_x_, T_p1_, T_p2_of the Al_70_Fe_12.5_V_12.5_Nb_5_ amorphous alloy. (**a**) Kissinger plot, (**b**) Ozawa plot.

Figure 4 reports the Kissinger and Ozawa plots by linear fitting of ln (β/T^2^) versus 1/T or ln (β) versus 1/T. The estimated activation energies (E_x_, E_p1_, E_p2_) corresponding to T_x_, T_p1_, T_p2_ were evaluated by the Kissinger and Ozawa methods, and listed in Table 2. The fitted curves are close to the straight lines; the Adjusted R-square is higher than 96, and the results of the two equations have the same tendency, which proves the reliability of the data. It is clear that the effective activation energies evaluated by the Kissinger equation are smaller than Ozawa equation. The E_x_ values resulting by the application of the Kissinger and Ozawa methods can be calculated: 366.3 ± 23.9 and 380.5 ± 23.9 kJ/mol, respectively.

**Figure 5:**
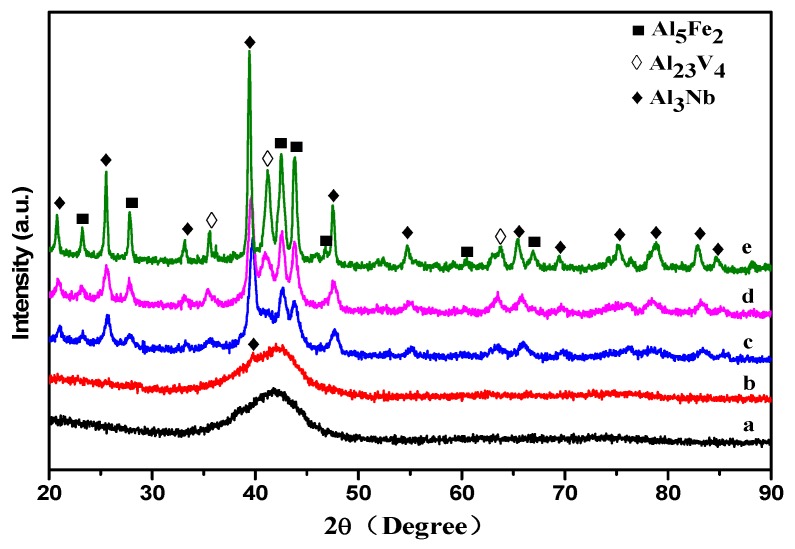
XRD patterns of the Al_70_Fe_12.5_V_12.5_Nb_5_ amorphous alloy powders after heat treatment: (a) fast cooling after heating to 773 K; (b) annealing at 773 K for 100 min; (c) fast cooling after heating to 973 K; (d) annealing at 973 K for 100 min; (e) fast cooling after heating to 1173 K.

The DSC curves in Figure 3 have two obvious endothermic peaks, which means that there are two crystallization processes. To further understand the crystallization of the new crystalline phases during the exothermic processes, five heat treatments of the Al_70_Fe_12.5_V_12.5_Nb_5_ amorphous alloy powders were studied. Figure 5 shows the XRD pattern of the Al_70_Fe_12.5_V_12.5_Nb_5_ amorphous alloy powders after five different heat treatments. Different heat treatment temperatures have been selected: 773 K (below T_x_ and above T_g_), 973 K (between T_P1_ and T_P2_), and 1173 K (above T_P2_).

The crystal phase is not observed when the sample has been heated to 773 K before rapid cooling (heat treatment *a* in Figure 5). When amorphous alloy samples are annealed at 773 K for 100 min (heat treatment *b*), a few Al_3_Nb phases of the amorphous sample can be observed. It is shown that the short-range atom structure retained in the amorphous alloy has been partly changed after low-temperature annealing, while a certain degree of relaxation has occurred. Although the crystallization temperature has not been reached, the atom diffusion ability of the amorphous alloy has increased after annealing for 100 min with the Nb addition. Al_5_Fe_2_ and Al_3_Nb phases have been mainly observed after initial crystallization and rapid cooling immediately after heating to 973 K (heat treatment *c*). The Al_23_V_4_ phase is also found at 973 K annealing for 100 min (heat treatment *d*) and heating to 1173 K (heat treatment *e*). New crystalline phases precipitate when the amorphous sample is annealed, which may be due to the relaxation of the amorphous structure. To reduce the internal free energy of the amorphous alloy, a certain crystallization phase has precipitated in the amorphous alloy.

## 4. Conclusions

Amorphous alloy Al_70_Fe_12.5_V_12.5_Nb_5_ (at %) powders were successfully prepared via MA. The replacement of Al with Nb in the Al_75_V_12.5_Fe_12.5_ amorphous alloy effectively enhances its glass forming ability and thermal stability. The Al_70_Fe_12.5_V_12.5_Nb_5_ amorphous alloy exhibits a larger supercooled liquid region than 100 K, higher GFA with the T_rg_ of 0.522, and γ of 0.398 (20 K/min). The crystallization of the Al_70_Fe_12.5_V_12.5_Nb_5_ amorphous alloy takes place in two distinct steps, as observed via DSC analyses. Our study provides deeper insights into the development of Al-base amorphous alloys to be used in powder metallurgy industry.

## Figures and Tables

**Table 1:** The thermal properties and ΔT_x_, T_rg_, γ of Al_70_Fe_12.5_V_12.5_Nb_5_ measured at 10, 20, 30, and 40 K/min heating rates.

β(K/min)	T_g_(K)	T_x_ (K)	T_p1_ (K)	T_p2_ (K)	T_m_(K)	T_l_ (K)	ΔT_x_(K)	T_rg_	γ
10	726.7	844.2	866.2	1097.7	1375.2	1408.7	117.5	0.516	0.395
20	736.9	854.1	881.0	1112.4	1377.9	1407.9	117.2	0.522	0.398
30	749.8	862.5	888.8	1120.1	1377.5	1416.9	112.7	0.529	0.398
40	759.0	865.5	890.9	1125.7	1376.5	1427.3	106.5	0.532	0.396

**Table 2:** Energies of E_x_, E_p1_, E_p2_ estimated with the Kissinger and Ozawa methods.

Equations	E_x_ (KJ/mol)	E_p1_ (KJ/mol)	E_p2_ (KJ/mol)
Kissinger	366.3 ± 23.9	326.2 ± 35.0	489.4 ± 12.2
Ozawa	380.5 ± 23.9	340.8 ± 35.0	507.8 ± 12.2
